# 1,3-Di-*n*-butyl­thio­urea

**DOI:** 10.1107/S1600536811009743

**Published:** 2011-03-19

**Authors:** Andrzej Okuniewski, Agnieszka Dąbrowska, Jaroslaw Chojnacki

**Affiliations:** a Department of Inorganic Chemistry, Gdansk University of Technology, 11/12 Narutowicza Str., 80-233 Gdańsk, Poland

## Abstract

In the title compound, C_9_H_20_N_2_S, the *n*-butyl groups are in *syn* and *anti* positions in relation to the C=S bond. In the crystal, two mol­ecules are connected by two N—H⋯S=C hydrogen bonds into a centrosymmetric dimer. Another N—H⋯S=C hydrogen bond links the dimers, forming layers with a hydro­philic inter­ior and a hydro­phobic exterior, which spread across the (100) plane. Inter­lacing of the external butyl groups combines these layers into a three-dimensional structure.

## Related literature

For structures of *N*,*N′*-di-*n*-butyl­thio­urea complexes with mercury and copper, see: Ahmad *et al.* (2009 ▶); Khan *et al.* (2007 ▶); Warda (1998 ▶). For structures of other symmetrically substituted thio­urea derivatives, see: Custelcean *et al.* (2005 ▶); Djurdjevic *et al.* (2007 ▶); Ramnathan *et al.* (1995 ▶). For synthetic methods, see: Herr *et al.* (2000 ▶); Kricheldorf (1970 ▶); Ranu *et al.* (2003 ▶). 
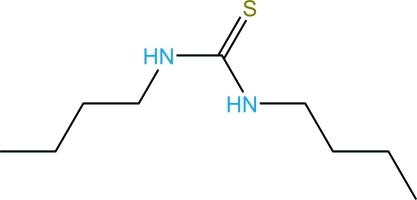


## Experimental

### 

#### Crystal data

C_9_H_20_N_2_S
*M* *_r_* = 188.33
Monoclinic, 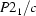

*a* = 12.6395 (6) Å
*b* = 10.0836 (6) Å
*c* = 9.0128 (5) Å
β = 90.476 (5)°
*V* = 1148.66 (11) Å^3^
*Z* = 4
Mo *K*α radiation
μ = 0.24 mm^−1^
*T* = 120 K
0.48 × 0.29 × 0.09 mm

#### Data collection

Oxford Diffraction Xcalibur Sapphire2 diffractometer
Absorption correction: analytical [*CrysAlis PRO* (Oxford Diffraction, 2010 ▶; based on Clark & Reid, 1995 ▶)] *T*_min_ = 0.94, *T*_max_ = 0.978
5268 measured reflections
2247 independent reflections
1656 reflections with *I* > 2σ(*I*)
*R*_int_ = 0.038

#### Refinement

*R*[*F* ^2^ > 2σ(*F* ^2^)] = 0.046
*wR*(*F* ^2^) = 0.116
*S* = 0.97
2247 reflections
119 parameters
2 restraints
H atoms treated by a mixture of independent and constrained refinement
Δρ_max_ = 0.45 e Å^−3^
Δρ_min_ = −0.27 e Å^−3^

### 

Data collection: *CrysAlis PRO* (Oxford Diffraction, 2010 ▶); cell refinement: *CrysAlis PRO*; data reduction: *CrysAlis PRO*; program(s) used to solve structure: *SHELXS97* (Sheldrick, 2008 ▶); program(s) used to refine structure: *SHELXL97* (Sheldrick, 2008 ▶); molecular graphics: *OLEX2* (Dolomanov *et al.*, 2009 ▶) and *Mercury* (Macrae *et al.*, 2008 ▶); software used to prepare material for publication: *WinGX* (Farrugia, 1999 ▶) and *PLATON* (Spek, 2009 ▶).

## Supplementary Material

Crystal structure: contains datablocks global, I. DOI: 10.1107/S1600536811009743/si2344sup1.cif

Structure factors: contains datablocks I. DOI: 10.1107/S1600536811009743/si2344Isup2.hkl

Additional supplementary materials:  crystallographic information; 3D view; checkCIF report

## Figures and Tables

**Table 1:** Hydrogen-bond geometry (Å, °)

*D*—H⋯*A*	*D*—H	H⋯*A*	*D*⋯*A*	*D*—H⋯*A*
N1—H1⋯S1^i^	0.84 (1)	2.58 (1)	3.3943 (17)	164 (2)
N2—H2⋯S1^ii^	0.85 (1)	2.52 (1)	3.3319 (17)	159 (2)

Symmetry codes: (i) 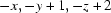; (ii) 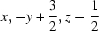.

**Fig. 1.**
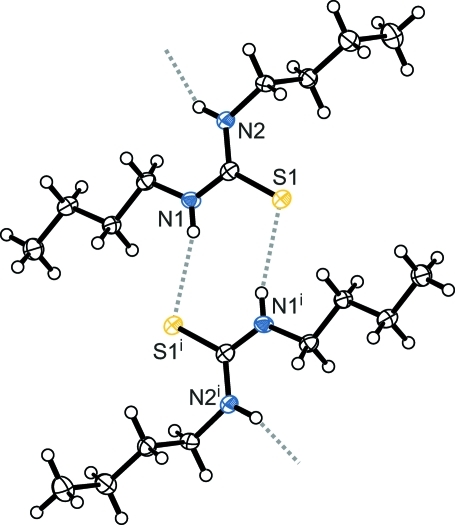
Structure of [SC(NHnBu)2]2 dimer with the thermal ellipsoids drawn at 50% probability level. Hydrogen bonds marked with dotted lines. Symmetry code (i): –x, –y + 1, –z + 2.

**Fig. 2.**
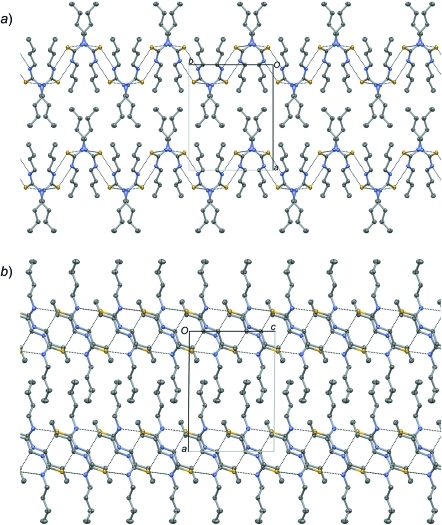
Layers of SC(NHnBu)2 seen in the a) [001] and b) [010] direction. Hydrogen bonds marked with dotted lines, hydrogen atoms omitted for clarity.

## supplementary crystallographic information

### Comment

*N*,*N'*-Di-*n*-butylthiourea, S═C(NH*^n^*Bu)_2_, is commonly used as a vulcanization accelerator in rubber processing, as an insecticide, as an additive to fertilizers, as a corrosion inhibitor, as an agent for metal treatments, *etc*.

No X-ray structure of pure compound was known until now, although the Cambridge Structural Database contains data on its mercury(ii) (Ahmad *et al.*, 2009), copper(i) (Khan *et al.*, 2007) and copper(ii) (Warda, 1998) complexes. In their structures one of the *n*-butyl groups of *N*,*N'*-di-*n*-butylthiourea molecule is in the *syn* position and the second is in the *anti* position in relation to the C═S bond. The same conformation is present in the title compound and this allows the formation of the centrosymmetric dimers (see Fig. 1) held together by two N1—H···S1^i^═C1^i^ hydrogen bonds [symmetry code: (i): –*x*, –*y* + 1, –*z* + 2]. Namely, motif R_2_^2^(8) is formed. Furthermore, there are additional N2—H···S1^ii^═C1^ii^ hydrogen bonds [symmetry code: (ii): *x*, –*y* + 3/2, *z*–1/2], which link the dimers to form the two-dimensional layers (see Fig. 2). Hydrogen bonds parameters are summarized in Tab. 1. Hydrocarbon chains pointing outside the layer interact with those from the neighbouring ones by van der Waals forces to form a three-dimensional crystal structure. The same packing patterns can be found in *syn*,*anti* isomers of other analogues: *N*,*N'*-diethylthiourea and *N*,*N'*-diisopropylthiourea (Ramnathan *et al.*, 1995) and similar in *N*,*N'*-bis(prop-2-en-1-yl)thiourea (Djurdjevic *et al.*, 2007). The case of *N*,*N'*-di-*tert*-butylthiourea is different, because the molecules adopt *syn*,*syn* conformation (Custelcean *et al.*, 2005).

There are several synthetic methods to obtain symmetrical thioureas. For example condensation of amine hydrochlorides with potassium thiocyanate (Herr *et al.*, 2000) or reaction of *N*-silylated amines with carbon disulfide (Kricheldorf, 1970). The very simple, quick and solvent-free method was proposed by Ranu *et al.* (2003) incorporating addition of amines to carbon disulfide on the surface of alumina under microwave irradition. In the case of *n*-butylamine, mixture was irradiated for 2 minutes and the yield was 89%.

We found that good quality crystals can be obtained by recrystallization from ethyl acetate or acetylacetone (2,4-pentanedione).

### Experimental

0.25 g (1.33 mmol) of commercially available *N*,*N'*-di-*n*-butylthiourea was dissolved in 2 ml of freshly distilled acetylacetone. The mixture was filtered and the filtrate was left for crystallization in a refrigerator. After several days well formed, colorless crystals were collected. Melting point: 335 - 337 K.

### Refinement

Hydrogen atoms were placed at the calculated positions (*d*_CH_ = 0.98–0.99 Å) and were treated as riding on their parent atoms, with *U*_iso_(H) set to 1.2–1.5 times *U*_eq_(C). The N—H distances were restrained to 0.85 (1) Å.

### Crystal data

**Table:**

C_9_H_20_N_2_S	*F*(000) = 416
*M**_r_* = 188.33	*D*_x_ = 1.089 Mg m^−^^3^
Monoclinic, *P*2_1_/*c*	Melting point: 336(1) K
Hall symbol: -P 2ybc	Mo *K*α radiation, λ = 0.71073 Å
*a* = 12.6395 (6) Å	Cell parameters from 2942 reflections
*b* = 10.0836 (6) Å	θ = 2.6–28.6°
*c* = 9.0128 (5) Å	µ = 0.24 mm^−^^1^
β = 90.476 (5)°	*T* = 120 K
*V* = 1148.66 (11) Å^3^	Prism, clear colourless
*Z* = 4	0.48 × 0.28 × 0.09 mm

### Data collection

**Table:**

Oxford Diffraction Xcalibur Sapphire2 diffractometer	2247 independent reflections
graphite	1656 reflections with *I* > 2σ(*I*)
Detector resolution: 8.1883 pixels mm^-1^	*R*_int_ = 0.038
ω scans	θ_max_ = 26°, θ_min_ = 2.6°
Absorption correction: analytical [*CrysAlis PRO* (Oxford Diffraction, 2010; based on Clark & Reid, 1995)]	*h* = −15→15
*T*_min_ = 0.94, *T*_max_ = 0.978	*k* = −12→12
5268 measured reflections	*l* = −10→11

### Refinement

**Table:**

Refinement on *F*^2^	Primary atom site location: structure-invariant direct methods
Least-squares matrix: full	Secondary atom site location: difference Fourier map
*R*[*F*^2^ > 2σ(*F*^2^)] = 0.046	Hydrogen site location: inferred from neighbouring sites
*wR*(*F*^2^) = 0.116	H atoms treated by a mixture of independent and constrained refinement
*S* = 0.97	*w* = 1/[σ^2^(*F*_o_^2^) + (0.0753*P*)^2^] where *P* = (*F*_o_^2^ + 2*F*_c_^2^)/3
2247 reflections	(Δ/σ)_max_ < 0.001
119 parameters	Δρ_max_ = 0.45 e Å^−^^3^
2 restraints	Δρ_min_ = −0.27 e Å^−^^3^

### Special details

**Table:**

Experimental. CrysAlisPro, Oxford Diffraction Ltd., Version 1.171.33.66 (Oxford Diffraction, 2010) Analytical numeric absorption correction using a multifaceted crystal model based on expressions derived by Clark & Reid (1995).
Geometry. All s.u.'s (except the s.u. in the dihedral angle between two l.s. planes) are estimated using the full covariance matrix. The cell s.u.'s are taken into account individually in the estimation of s.u.'s in distances, angles and torsion angles; correlations between s.u.'s in cell parameters are only used when they are defined by crystal symmetry. An approximate (isotropic) treatment of cell s.u.'s is used for estimating s.u.'s involving l.s. planes.
Refinement. Refinement of *F*^2^ against ALL reflections. The weighted *R*-factor *wR* and goodness of fit *S* are based on *F*^2^, conventional *R*-factors *R* are based on *F*, with *F* set to zero for negative *F*^2^. The threshold expression of *F*^2^ > 2σ(*F*^2^) is used only for calculating *R*-factors(gt) *etc*. and is not relevant to the choice of reflections for refinement. *R*-factors based on *F*^2^ are statistically about twice as large as those based on *F*, and *R*- factors based on ALL data will be even larger.

### Fractional atomic coordinates and isotropic or equivalent isotropic displacement parameters (Å^2^)

**Table:**

	*x*	*y*	*z*	*U*_iso_*/*U*_eq_	
S1	0.16510 (4)	0.55610 (5)	1.03064 (5)	0.02246 (18)	
N1	0.03660 (13)	0.60898 (18)	0.80521 (17)	0.0218 (4)	
N2	0.19164 (13)	0.72585 (17)	0.80620 (18)	0.0229 (4)	
C1	0.12922 (15)	0.63626 (19)	0.8705 (2)	0.0209 (4)	
C2	−0.00052 (15)	0.6657 (2)	0.6656 (2)	0.0225 (5)	
H2A	0.0525	0.649	0.5877	0.027*	
H2B	−0.0082	0.7629	0.6769	0.027*	
C3	−0.10604 (15)	0.6065 (2)	0.6181 (2)	0.0232 (5)	
H3A	−0.1004	0.5086	0.6174	0.028*	
H3B	−0.1608	0.6314	0.6909	0.028*	
C4	−0.13954 (16)	0.6542 (2)	0.4653 (2)	0.0260 (5)	
H4A	−0.0831	0.6327	0.3938	0.031*	
H4B	−0.1475	0.7518	0.4676	0.031*	
C5	−0.24282 (17)	0.5926 (2)	0.4116 (2)	0.0332 (5)	
H5A	−0.2359	0.4959	0.4103	0.05*	
H5B	−0.259	0.6244	0.3112	0.05*	
H5C	−0.3001	0.618	0.4785	0.05*	
C6	0.29696 (15)	0.7629 (2)	0.8598 (2)	0.0239 (5)	
H6A	0.296	0.7677	0.9695	0.029*	
H6B	0.3145	0.8523	0.8218	0.029*	
C7	0.38310 (15)	0.6662 (2)	0.8128 (2)	0.0254 (5)	
H7A	0.365	0.5764	0.8489	0.03*	
H7B	0.3855	0.6629	0.7031	0.03*	
C8	0.49139 (17)	0.7044 (2)	0.8722 (3)	0.0355 (6)	
H8A	0.489	0.7081	0.9819	0.043*	
H8B	0.5097	0.7941	0.8357	0.043*	
C9	0.57675 (19)	0.6074 (3)	0.8255 (3)	0.0469 (7)	
H9A	0.5583	0.5182	0.8596	0.07*	
H9B	0.6446	0.634	0.8697	0.07*	
H9C	0.5824	0.6075	0.7172	0.07*	
H1	−0.0032 (14)	0.5586 (19)	0.854 (2)	0.028 (6)*	
H2	0.1715 (15)	0.7675 (18)	0.7292 (15)	0.019 (6)*	

### Atomic displacement parameters (Å^2^)

**Table:**

	*U*^11^	*U*^22^	*U*^33^	*U*^12^	*U*^13^	*U*^23^
S1	0.0254 (3)	0.0207 (3)	0.0213 (3)	0.0005 (2)	0.00074 (19)	0.0018 (2)
N1	0.0218 (9)	0.0250 (9)	0.0188 (8)	−0.0043 (8)	0.0011 (7)	0.0019 (7)
N2	0.0238 (9)	0.0227 (9)	0.0221 (9)	−0.0016 (7)	−0.0019 (7)	0.0047 (7)
C1	0.0251 (10)	0.0180 (10)	0.0197 (10)	0.0039 (8)	0.0051 (8)	−0.0024 (8)
C2	0.0257 (10)	0.0213 (11)	0.0206 (10)	0.0003 (9)	0.0037 (8)	0.0015 (8)
C3	0.0234 (10)	0.0223 (10)	0.0238 (11)	−0.0007 (8)	0.0013 (8)	0.0018 (8)
C4	0.0325 (12)	0.0223 (11)	0.0232 (11)	−0.0027 (9)	−0.0003 (9)	0.0014 (8)
C5	0.0325 (12)	0.0355 (13)	0.0313 (12)	−0.0036 (10)	−0.0068 (10)	0.0028 (10)
C6	0.0226 (10)	0.0219 (11)	0.0272 (11)	−0.0047 (9)	−0.0010 (8)	0.0011 (8)
C7	0.0251 (11)	0.0280 (11)	0.0230 (10)	0.0000 (9)	−0.0003 (8)	0.0020 (9)
C8	0.0276 (12)	0.0329 (13)	0.0460 (14)	−0.0016 (10)	−0.0010 (10)	0.0020 (11)
C9	0.0292 (13)	0.0475 (16)	0.0641 (18)	0.0056 (12)	0.0016 (12)	0.0049 (14)

### Geometric parameters (Å, °)

**Table:**

S1—C1	1.712 (2)	C5—H5A	0.98
N1—C1	1.334 (2)	C5—H5B	0.98
N1—C2	1.456 (2)	C5—H5C	0.98
N1—H1	0.844 (9)	C6—C7	1.524 (3)
N2—C1	1.335 (3)	C6—H6A	0.99
N2—C6	1.461 (3)	C6—H6B	0.99
N2—H2	0.849 (9)	C7—C8	1.515 (3)
C2—C3	1.519 (3)	C7—H7A	0.99
C2—H2A	0.99	C7—H7B	0.99
C2—H2B	0.99	C8—C9	1.518 (3)
C3—C4	1.515 (3)	C8—H8A	0.99
C3—H3A	0.99	C8—H8B	0.99
C3—H3B	0.99	C9—H9A	0.98
C4—C5	1.521 (3)	C9—H9B	0.98
C4—H4A	0.99	C9—H9C	0.98
C4—H4B	0.99		
			
C1—N1—C2	125.10 (17)	H5A—C5—H5B	109.5
C1—N1—H1	114.7 (15)	C4—C5—H5C	109.5
C2—N1—H1	120.1 (15)	H5A—C5—H5C	109.5
C1—N2—C6	124.74 (17)	H5B—C5—H5C	109.5
C1—N2—H2	121.0 (14)	N2—C6—C7	113.32 (17)
C6—N2—H2	114.2 (14)	N2—C6—H6A	108.9
N1—C1—N2	117.90 (18)	C7—C6—H6A	108.9
N1—C1—S1	119.96 (15)	N2—C6—H6B	108.9
N2—C1—S1	122.13 (15)	C7—C6—H6B	108.9
N1—C2—C3	111.42 (16)	H6A—C6—H6B	107.7
N1—C2—H2A	109.3	C8—C7—C6	112.61 (18)
C3—C2—H2A	109.3	C8—C7—H7A	109.1
N1—C2—H2B	109.3	C6—C7—H7A	109.1
C3—C2—H2B	109.3	C8—C7—H7B	109.1
H2A—C2—H2B	108	C6—C7—H7B	109.1
C4—C3—C2	111.70 (16)	H7A—C7—H7B	107.8
C4—C3—H3A	109.3	C7—C8—C9	112.3 (2)
C2—C3—H3A	109.3	C7—C8—H8A	109.1
C4—C3—H3B	109.3	C9—C8—H8A	109.1
C2—C3—H3B	109.3	C7—C8—H8B	109.1
H3A—C3—H3B	107.9	C9—C8—H8B	109.1
C3—C4—C5	113.14 (17)	H8A—C8—H8B	107.9
C3—C4—H4A	109	C8—C9—H9A	109.5
C5—C4—H4A	109	C8—C9—H9B	109.5
C3—C4—H4B	109	H9A—C9—H9B	109.5
C5—C4—H4B	109	C8—C9—H9C	109.5
H4A—C4—H4B	107.8	H9A—C9—H9C	109.5
C4—C5—H5A	109.5	H9B—C9—H9C	109.5
C4—C5—H5B	109.5		
			
C2—N1—C1—N2	2.5 (3)	N1—C2—C3—C4	−173.97 (16)
C2—N1—C1—S1	−176.99 (15)	C2—C3—C4—C5	177.74 (17)
C6—N2—C1—N1	−177.36 (17)	C1—N2—C6—C7	81.5 (2)
C6—N2—C1—S1	2.1 (3)	N2—C6—C7—C8	−178.71 (17)
C1—N1—C2—C3	176.27 (17)	C6—C7—C8—C9	179.74 (19)

### Hydrogen-bond geometry (Å, °)

**Table:**

*D*—H···*A*	*D*—H	H···*A*	*D*···*A*	*D*—H···*A*
N1—H1···S1^i^	0.84 (1)	2.58 (1)	3.3943 (17)	164 (2)
N2—H2···S1^ii^	0.85 (1)	2.52 (1)	3.3319 (17)	159 (2)

Symmetry codes: (i) −*x*, −*y*+1, −*z*+2; (ii) *x*, −*y*+3/2, *z*−1/2.
